# Not urbanization level but socioeconomic, physical and social neighbourhood characteristics are associated with presence and severity of depressive and anxiety disorders

**DOI:** 10.1017/S0033291718000612

**Published:** 2018-03-15

**Authors:** Ellen Generaal, Erik J. Timmermans, Jasper E. C. Dekkers, Johannes H. Smit, Brenda W. J. H. Penninx

**Affiliations:** 1 Department of Psychiatry, Amsterdam Public Health Research Institute, VU University Medical Center, Amsterdam, the Netherlands; 2 Department of Epidemiology and Biostatistics, Amsterdam Public Health Research Institute, VU University Medical Center, Amsterdam, the Netherlands; 3 Spatial Information Laboratory, Department of Spatial Economics, Faculty of Economics and Business Administration, VU University Amsterdam, Amsterdam, the Netherlands

**Keywords:** Anxiety, cohort studies, depression, environment, environment and public health, mental health, neighbourhood, residence characteristics, social environment

## Abstract

**Background:**

Which neighbourhood factors most consistently impact on depression and anxiety remains unclear. This study examines whether objectively obtained socioeconomic, physical and social aspects of the neighbourhood in which persons live are associated with the presence and severity of depressive and anxiety disorders.

**Methods:**

Cross-sectional data are from the Netherlands Study of Depression and Anxiety including participants (*n* = 2980) with and without depressive and anxiety disorders in the past year (based on DSM-based psychiatric interviews). We also determined symptom severity of depression (Inventory of Depression Symptomatology), anxiety (Beck Anxiety Inventory) and fear (Fear Questionnaire). Neighbourhood characteristics comprised socioeconomic factors (socioeconomic status, home value, number of social security beneficiaries and percentage of immigrants), physical factors (air pollution, traffic noise and availability of green space and water) and social factors (social cohesion and safety). Multilevel regression analyses were performed with the municipality as the second level while adjusting for individual sociodemographic variables and household income.

**Results:**

Not urbanization grade, but rather neighbourhood socioecononomic factors (low socioeconomic status, more social security beneficiaries and more immigrants), physical factors (high levels of traffic noise) and social factors (lower social cohesion and less safety) were associated with the presence of depressive and anxiety disorders. Most of these neighbourhood characteristics were also associated with increased depressive and anxiety symptoms severity.

**Conclusion:**

These findings suggest that it is not population density in the neighbourhood, but rather the quality of socioeconomic, physical and social neighbourhood characteristics that is associated with the presence and severity of affective disorders.

## Introduction

The role of neighbourhood characteristics in health research is receiving growing attention over the past 20 years (Arcaya *et al.* 2016). Besides impacts on physical health (Diez Roux & Mair, 2010), an increasing amount of studies also focused on the potential psychological impacts of the living environment (Truong & Ma, 2006; Peen *et al.* 2010; Gong *et al.* 2016). Urbanization and socioeconomic, physical and social neighbourhood characteristics might be associated with mental health through environmental stressors, such as overcrowding, violence and low social cohesion and social support (Evans, 2003; Srivastava, 2009; Blair *et al.* 2014; van Deurzen *et al.* 2016). In addition, the neighbourhood might relate to mental health through their an unfavourable impact on biological stress regulation pathways, such as those involved in immune (Calderón-Garcidueñas *et al.* 2008; Chen *et al.* 2017) or HPA-axis (Dulin-Keita *et al.* 2012; Hackman *et al.* 2012) regulation. However, which neighbourhood factors are most consistently associated with depressive and anxiety disorders remains rather unclear, mainly because previous studies are heterogeneous in their measurement of psychiatric outcomes, neighbourhood factors and confounders (Truong & Ma, 2006; Diez Roux, 2007; Peen *et al.* 2010; Gong *et al.* 2016).

Studies found higher prevalences for clinical depressive and anxiety disorders in urban areas as compared to rural areas in several developed countries (Peen *et al.* 2010; Zijlema *et al.* 2015). However, a recent study showed that increased urbanization was not significantly associated with DSM-based depressive and anxiety disorders, but only with self-reported indicators of mental health (de Vries *et al.* 2016). Regarding socioeconomic neighbourhood characteristics, both person-level and neighbourhood-level income status has previously been associated with depressive symptoms, depressive disorders and major depressive episodes (Kim, 2008; Annequin *et al.* 2015; Klijs *et al.* 2016).

Previous studies have also covered the physical neighbourhood in relation to depression and anxiety outcomes (Miles *et al.* 2012; de Vries *et al.* 2016; Zijlema *et al.* 2016*a*, *b*). Some used subjective measures of ‘perception’ of the physical neighbourhood (Ruijsbroek *et al.* 2017) whereas others used objective census data (Miles *et al.* 2012; de Vries *et al.* 2016), methods which have shown to yield different results (Ball *et al.* 2008; Gebel *et al.* 2011; Linden & Vodermaier, 2012). One recent multi-cohort study found inconsistent evidence for an association of subjective reports of more green space and better green space quality with lower depressive symptoms among four European countries (Ruijsbroek *et al.* 2017). Another study showed that census-data-based green space availability was associated with a lower prevalence of anxiety disorders, but not with depressive disorders (de Vries *et al.* 2016). In that study, more water availability in the neighbourhood was associated with the presence of both anxiety and depressive disorders (de Vries *et al.* 2016). Regarding other physical neighbourhood factors, air pollution has consistently been associated with physical health outcomes, such as pulmonary disease (Gehring *et al.* 2013) and cardiovascular disease (Cesaroni *et al.* 2014), but associations with affective disorders are less clear and absent in a recent European study (Zijlema *et al.* ^103,105^). Similarly, traffic noise has frequently been associated with physical outcomes such as high heart rate (Zijlema *et al.* 2016*a*), but inconclusive evidence was found for an association with depressive and anxiety symptoms, as indicated in a recent systematic review (Tzivian *et al.* 2015).

The association between the social neighbourhood and depression and anxiety also remains to be elucidated, as most previous studies measured the subjective experience of social environmental factors, such as safety and poor social interactions (Mair *et al.* 2008; Ruijsbroek *et al.* 2016). This might result in bias because poor mental health may change perception and influence self-report of the environment (Diez Roux & Mair, 2010). This underlines the need of objectively measured neighbourhood factors by using national registration systems. Besides this, most previous studies (Kim, 2008; Mair *et al.* 2008; Diez Roux & Mair, 2010; Zijlema *et al.* 2015; de Vries *et al.* 2016; Klijs *et al.* 2016) were limited in examining only one aspect of the neighbourhood even though neighbourhood factors are highly intercorrelated. Large-scale multivariable studies are needed to test whether associations still hold when adjusting for other relevant neighbourhood factors such as urbanization grade and for individual indicators of socioeconomic status (Kim, 2008). Strengths of the current study are (1) the inclusion of objective measures of a wide range of neighbourhood characteristics, (2) the assessment of depression and anxiety using semi-structured interviews according to DSM standards (Robins *et al.* 1988) and (3) the comparison with results from depression and anxiety symptom scales.

This large-scale cross-sectional study (*N* = 2980) examines to what extent (1) urbanization grade; (2) neighbourhood socioeconomic characteristics, i.e. socioeconomic status, home value, number of social security beneficiaries and percentage of immigrants; (3) neighbourhood physical characteristics, i.e. air pollution, traffic noise and availability of green space and water; and (4) neighbourhood social characteristics, i.e. social cohesion and safety, are (independently) associated with the presence and severity of depressive and/or anxiety disorders, while adjusting for individual socioeconomic variables.

## Methods

### Sample

The current cross-sectional study used data from the Netherlands Study of Depression and Anxiety (NESDA): an ongoing longitudinal cohort study in which 2981 participants (18–65 years at baseline) were followed to investigate the long-term course and consequences of depressive and anxiety disorders. To establish inclusion of patients at different developmental stages of their disorder, participants were recruited from the community (19%), from primary care (54%) and from specialized mental health care (27%). At baseline (2004–2007), persons with a range of psychopathology were included: from those without a depressive or anxiety disorder to those with a current, first or recurrent (in the past year) depressive or anxiety disorder and those with a remitted disorder (at baseline, a depressive and/or anxiety disorder was diagnosed in the past, but no diagnoses were present in the year before baseline). Exclusion criteria were not being fluent in Dutch and a primary clinically overt diagnosis of other psychiatric (e.g. diagnosis of psychotic, obsessive-compulsive, bipolar or severe addiction) disorder. Most NESDA participants were recruited from three different regions in the Netherlands, surrounding the cities of Amsterdam (north-west of the Netherlands), Leiden (mid-west) or Groningen (north-east). These cities have a variety of urbanization (cities with ±800.000, ±120.000 and ±200.000 habitants, respectively), and also persons from the larger rural areas around these cities were recruited. As compared with the whole Netherlands, NESDA included more persons from the extremely and strongly urbanized areas (65% *v.* 40%) and less from the hardly and not urbanized areas (16% *v.* 42%). Nonetheless, these more rural areas are still well represented in NESDA (±16% equals *n* = 481) and there is a rather large variation in urbanization grades within our study, which is comparable to the entire Netherlands. In NESDA, we included subjects from 666 different postal code areas (out of a total of ±4000 four-digit postal code areas in the Netherlands; CBS, 2006*f*) and from 200 different municipalities (out of a total of 458 municipalities in the Netherlands; CBS, 2005). On average, 25 participants live in one postal code area (range = 1–105), and 338 participants live in one municipality (range 1–787) within our study.

The research protocol was approved by the Ethical Committee of participating universities and written informed consent was obtained from all participants. Penninx *et al.* provide a detailed description of the NESDA study design and sampling procedures (Penninx *et al.* 2008).

Data for the current study were available of 2980 participants due to a failure to administer one postal code by the staff. Due to item missing values, the sample varied from *n* = 2966 [for a socioeconomic status score (SES)] to *n* = 2980 (for urbanization grade, home value and percentage of immigrants).

### Presence and severity of depressive and anxiety disorders

The presence of current diagnoses of depressive disorders (major depressive disorder and dysthymia) and anxiety disorders (panic disorder, agoraphobia, generalized anxiety disorder and social phobia) were determined in the year prior to baseline interview using the semi-structured Composite International Diagnostic Interview (WHO version 2.1) according to DSM-IV criteria (Robins *et al.* 1988). Controls were defined as individuals without current diagnoses of depressive and anxiety disorders.

Severity of depressive and anxiety disorders were measured using the 28-item Inventory of Depression Symptomatology (IDS, range 0–84) (Rush *et al.* 1996), the 21-item Beck Anxiety Inventory (BAI, range 0–63) (Beck *et al.* 1988) and the 15-item Fear Questionnaire (FQ, range 0–120) (Marks & Mathews, 1979). The IDS measures the overall severity of depression in the past week, whereas the BAI assesses the overall severity of anxiety in the past week, e.g. feeling nervous, shaky or dizzy. The FQ reflects the level of avoidance (0 = ‘never avoid it’ to 8 = ‘always avoid it’) related to symptoms of social phobia, blood phobia and agoraphobia, e.g. fear of talking to people in authority, fear of injections and fear of large public spaces. Psychometric properties for the IDS (IDS, 2017), the BAI (Beck *et al.* 1988; Beck & Steer, 1990) and the FQ (van Zuuren, 1988) have been well established.

### Neighbourhood factors

Data of neighbourhood factors were retrieved from national registration organizations [e.g. Statistics Netherlands (CBS)]. Details have been described elsewhere (Timmermans *et al.*, 2018; CBS, 2006*a*, *g*). All neighbourhood factors were aggregated to mean values for each four-digit postal code area and subsequently linked to NESDA cohort data using respondents’ four-digit postal codes at baseline assessment. For all neighbourhood factors, data were used of the year 2006 (CBS, 2006*a*, *c*), with the exception of noise (2007) and air pollution (2009) as those were unavailable in 2006. Baseline assessment of NESDA took place between September 2004 and February 2007, and 89% of our sample were interviewed in 2005 or 2006.

Correlations of neighbourhood factors within each domain are moderate to high (see online Supplementary Table 1). The range in correlations were *r* =  −0.83 to −0.17 within the socioeconomic domain; *r* =  −0.47 to 0.66 within the physical domain, and *r* = 0.61 within the social domain, suggesting that all neighbourhood factors partly represent differential aspects of neighbourhood quality. In addition, correlations of neighbourhood factors across domains are generally somewhat lower than those within one domain (see online Supplementary Table 1).

#### Urbanization grade

Urbanization grade is the mean number of addresses per squared kilometre within a circle with a radius of 1 km (den Dulk *et al.* 1992). Data are provided by Statistics Netherlands and are defined in five categories: (1) extremely urbanized (⩾2500 addresses/km^2^), (2) strongly urbanized (1500–2500 addresses/km^2^), (3) moderately urbanized (1000–1500 addresses/km^2^), (4) hardly urbanized (500–1000 addresses/km^2^) and (5) not urbanized (<500 addresses/km^2^).

#### Socioeconomic neighbourhood factors

SESs were obtained from the Netherlands Institute of Social Research (SCP, 2017). The SES score is based on three dimension scores: education, income and position in the labour market. A principal component analyses defined the SES score based on mean income, percentage low incomes, percentage of low educated residents and percentage of unemployed residents. More details are available elsewhere (Knol, 2012; SCP, 2017).

The average home value (in k€) was provided by Statistics Netherlands (CBS, 2017*a*). Residential housing and residential housing with (private) practices were included, whereas recreational housing and other non-residential housing such as garage boxes were excluded from the calculation of the score (CBS, 2006*c*; WOZ, 2017).

The number of social security beneficiaries per 1000 households was also provided by Statistics Netherlands (CBS, 2006*c*). Social security benefits in the Netherlands pertain to long-term unemployment, either due to disability or the inability to (find) work. We excluded benefits issued to residents who lived in (healthcare) institutions (CBS, 2006*c*, 2014).

The percentage of immigrants (from all continents) among all residents was provided by Statistics Netherlands (CBS, 2006*c*). Residents were considered as immigrants if they were born outside the Netherlands with one or both parents born outside the Netherlands (first generation) or if they were born inside the Netherlands and one or both parents were born abroad (second generation). This is the most widely accepted and valid assessment of ethnicity in the Netherlands (Stronks *et al.* 2009). More details are described elsewhere (CBS, 2006*c*, *d*).

#### Physical neighbourhood factors

Daily mean noise of road- rail- and air traffic for several years were modelled by the Netherlands Environmental Assessment Agency by using the Empara Noisetool with a resolution of 25 × 25 m (PBL, 2017). This study uses noise data of 2007. Noise is measured in Level day–evening–night (Lden) and is expressed in decibels [dB (A)]. The measure Lden accounts for the fact that noise in the evening and the night are more annoying than during the day. The average noise levels during the day (7–19 h), the evening (19–23 h) and the night (23–7 h) were calculated first and the levels of noise in the evening and the night are increased with 5 and 10 dB (A), respectively. Subsequently, the daily mean noise was calculated by dividing the noise levels during day, evening and night by 3. The modelling of road-, rail- and air traffic noise accounts for several factors, such as sound barriers, rail type and flight paths. The noise data were linked to all addresses that were included in the Register of Addresses and Building (BAG-register, June 2015) of the Netherlands’ Cadastre, Land Registry, and Mapping Agency, by using GeoDMS software (Object Vision BV, Amsterdam, the Netherlands). More details on the assessment of traffic noise are available elsewhere (PBL, 2017). Traffic noise data for the Netherlands appears highly correlated over the years 2000–2008 (all Pearsons’ *r* = 0.98–0.99, with the exception of 2008 with 2000–2007: Pearsons’ *r* = 0.82), suggesting that noise exposure in the neighbourhood is relatively stable over time.

Annual average air pollution concentrations at the subjects’ home addresses were estimated by land-use regression models for the year 2009 by the Institute for Risk Assessment Sciences as part of the European Study of Cohorts for Air Pollution Effects (ESCAPE-project), as described elsewhere (Eeftens *et al.* 2012; Beelen *et al.* 2013). The current study uses data of the annual average concentrations of the mean blackness of PM_2.5_ filters, which is a proxy for elemental carbon (soot), the dominant light absorbing substance. Previous studies from Europe support the stability of spatial contrasts in black carbon levels over periods of 10 years and more (Eeftens *et al.* 2011; Gulliver *et al.* 2011; Cesaroni *et al.* 2014).

The percentage of green space and blue space (water) of total land use was calculated using the Calculate Geometry-option in ArcGIS (ArcGIS, 2017) and the neighbourhood map from Statistics Netherlands (CBS, 2006*g*). Categories of green space included recreation (e.g. parks, gardens), agriculture and forest or nature (see CBS, 2006*b*). Recreational green space had a minimal size of either 0.1 hectare (ha) (garden complexes), 0.5 ha (sports fields) or 1 ha (e.g. parks or other recreational property) in order to be included (CBS, 2006*b*). For agriculture and forests/nature, this lower limit was 1 ha (CBS, 2006*b*). Blue space included inland water, sea and (large) lakes. Minimal size were either 0 ha for large water areas (sea, rivers or big lakes were included independent of size) or 0.5 to 1 ha for other types of inland water (e.g. canals or lakes) and recreational water (e.g. ponds). For an exact description of green and blue space, we refer to CBS Soil Statistics (CBS, 2006*b*).

#### Social neighbourhood factors

Social cohesion and safety in the neighbourhood have been measured as part of the assessment of neighbourhood livability by the Netherlands Ministry of the Interior and Kingdom Relations (Leidelmeijer & van Kamp, 2003; Leidelmeijer *et al.* 2008; Livability scores, 2015). The social cohesion score (range, −50 to 50) is based on indicators such as a number of relocations and homogeneity of the family composition. The safety score (range, −50 to 50) is based on indicators such as annoyance (of drug abuse, loitering youth, neighbours, destruction, rubbish and daubing/graffiti) and number of crimes (Leefbaarometer, 2015).

### Covariates

Basic covariates included the following person-level variables: age, sex, years of education and household income (net € per month). These covariates are in line with previous studies on neighbourhood factors and mental health (de Vries *et al.* 2016; Zijlema *et al.* 2016*a*, *b*). The total income of the household was measured as a continuous variable (range, 1–24) with intervals of €200 for each value (i.e. 1 = ‘<€600’; 2 = ‘€600–800’; until 23 = ‘€4801–5000’; 24 = ‘>€5000’). In NESDA, one household comprises on average two persons, with a range from 1 to 10 persons (s.d. = 0.70). Missing values for baseline household income (*n* = 41) were imputed using NESDA follow-up data (first observation carried backward).

### Statistical analyses

Sample baseline characteristics were compared between subjects with and without depressive and/or anxiety disorders using independent-samples *t* tests for continuous variables, χ^2^ tests for dichotomous and categorical variables, and Mann–Whitney *U* tests for non-normally distributed variables. Descriptive analyses were computed in SPSS V.20 for Windows (SPSSinc, Chicago, USA).

Multilevel logistic and linear regression analyses were conducted to examine the associations between each neighbourhood factor with (1) the presence of current depressive and/or anxiety disorders, and (2) the severity of depressive, anxiety and fear symptoms (separate models) for participants (level 1), nested within municipalities (level 2). We first conducted main analyses for the presence of depression and/or anxiety *v.* controls, followed by analyses separately for depression and anxiety to test for differences across type of disorder. To take intra-neighbourhood correlation into account (Merlo *et al.* 2006), municipality code was included as a random intercept in the multilevel analyses, and this appeared of additive value (intraclass correlation coefficient = 0.15). Municipality codes/postal codes combinations were determined using CBS data of 2005 (CBS, 2005). Multilevel analyses were computed with R (version 3.3.2), using functions *glmer* and *lmer* from the ‘lme4’ package (version 1.1–12) (R software, 2017). Output of fixed parameter estimates were expressed as odds ratio for diagnoses of depression and anxiety, and as standardized regression coefficient (β) for severity of symptoms. Bootstrap confidence intervals were calculated (1000 bootstrap samples) using the ‘pbkrtest’ package (for lmer) and the Wald method of the *confint.merMod* function (for glmer). All multilevel analyses were adjusted for age, sex, years of education and household income. For all statistical tests, a probability level of ⩽5% was regarded as significant.

Separate multilevel regression analyses were conducted for each neighbourhood factor with the presence and severity of depression and anxiety. Subsequently, to test whether findings are confounded by other neighbourhood factors, we performed multivariable analyses including all neighbourhood factors. Variance inflation factors (Zuur *et al.* 2010) of multivariable models were below 4.3, which is considered acceptable for multicollinearity according to previous guidelines (Bowerman & O'Connell, 1990; Myers, 1990; Field, 2013).

To check whether our associations were affected by instability in home address of participants around the assessment period, we performed additional sensitivity analyses (1) excluding persons who lived at their home address for <2 years at baseline assessment (remaining *n* = 2485), and (2) excluding persons who moved between baseline and follow-up measurement in 2006–2009 (remaining *n* = 1986). Moreover, since the control group in our study (*n* = 1197) also includes individuals with remitted diagnoses of depressive and anxiety disorders, we performed additional analyses excluding persons from the control group who had experienced depressive and anxiety disorders earlier during their lifetime (remaining *n* = 2435 of which 652 controls).

## Results

Baseline characteristics of the study population are shown in Table 1. In subjects with current diagnoses of depressive and/or anxiety disorders, median score for depressive symptoms was 28 (IQR = 19–37), median score for anxiety symptoms was 15 (IQR = 9.0–23) and median score for fear symptoms was 30 (IQR = 16–46). Compared with controls, subjects with depression and/or anxiety were significantly younger, had less years of education and a lower household income (all *p* < 0.05). Individuals with depression/anxiety seem to live in neighbourhoods with lower socioeconomic status, higher levels of air pollution and higher levels traffic noise, more water and lower social cohesion as compared with controls (See Table 1: *p* < 0.05).

**Table 1.** Baseline characteristics^a^

	Control group	Current diagnoses of depression/anxiety		
	(*n* = 1197)	(*n* = 1783)	*P*	*N*
Sociodemographic factors				
Age (years)	43 (14)	41 (12)	0.004	2980
Women (%)	65	67	0.17	2980
Education (years)	13 (3.2)	12 (3.3)	<0.001	2980
Household income (€ net/month) (%)			<0.001	2973
Low (⩽1400 €)	21	30		
Medium (1401–2600 €)	37	39		
High (>2600 €)	43	31		
Neighbourhood characteristics				
*Urbanization grade (%)*			0.15	2980
Extreme (⩾2500 addresses/km^2^)	42	43		1262
Strong (1500–2500 addresses/km^2^)	24	22		679
Moderate (1000–1500 addresses/km^2^)	19	19		558
Hardly (500–1000 addresses/km^2^)	7.1	9.2		249
Not (<500 addresses/km^2^)	8.7	7.2		232
*Neighbourhood socioeconomic factors*				
Socioeconomic status score	0.16 (−0.18, 0.65)	0.04 (−0.97, 0.59)	0.02	2966
Home value (in k€)	198 (155, 244)	207 (157, 248)	0.12	2980
Social security beneficiaries (per 1000 households)	45 (27, 72)	45 (22, 82)	0.08	2975
Immigrants (%) in neighbourhood	20 (14, 32)	21 (13, 34)	0.21	2980
*Neighbourhood physical factors*				
Air pollution: PM_2.5_ absorbance (10^−5^ m^−1^)	1.36 (0.27)	1.38 (0.27)	0.006	2977
Road-, rail- and air-traffic noise (Lden, dB/24 hours)	55.2 (53.0, 56.8)	55.4 (53.4, 57.1)	0.02	2977
Green space in neighbourhood (% of land use)	18 (9.2, 40)	18 (9.0, 38)	0.27	2899
Water in neighbourhood (% of land use)	4.4 (1.3, 7.1)	4.5 (2.2, 8.0)	0.004	2899
*Neighbourhood social factors*				
Social cohesion score	−6.4 (−14, 2.6)	−7.8 (−15, 1.9)	0.007	2973
Safety score	−20 (−35, 4.5)	−21 (−40, 7.0)	0.07	2973
Clinical characteristics				
Severity of depressive symptoms	9.0 (4.0, 15)	28 (19, 37)	<0.001	2941
Severity of anxiety symptoms	4.0 (1.0, 8.0)	15 (9.0, 23)	<0.001	2945
Severity of fear symptoms	11 (4.0, 20)	30 (16, 46)	<0.001	2945

a Values are illustrated as mean (standard deviation; s.d.), median (interquartile range; IQR) or percentages (%). Household income is presented in categories for illustrative purposes; a continuous variable was used in the multilevel regression analyses. *N*-values could vary from total *N* = 2980 due to missings on individual measures.

### Neighbourhood characteristics and current diagnoses of depression and anxiety

Table 2 reports the associations of neighbourhood characteristics with current diagnoses of depressive disorders and anxiety disorders from multilevel logistic regression analyses (*N* = 2980). No significant associations were found for urbanization grade with depressive and anxiety disorders, neither in the main analyses nor in the analyses separately for depression and anxiety (*p* > 0.05). However, several socioeconomic, physical and social neighbourhood factors did appear associated with depressive and/or anxiety disorders (Table 2; *p* ⩽ 0.05). More specifically, neighbourhoods with low socioeconomic status (OR[95%CI] = 0.88[0.79–0.98]), a high number of social security beneficiaries [OR (95% CI) = 1.24 (1.11–1.39)], more immigrants (OR[95%CI] = 1.23(1.05, 1.44]), high levels of traffic noise [OR (95% CI) = 1.17 (1.03–1.32)], lower social cohesion [OR (95%CI) = 0.84 (0.76–0.93)] and less safety [OR (95% CI) = 0.84 (0.71–1.00)] were significantly associated with depressive disorders (Table 2, column 2; all *p* ⩽ 0.05). Of these factors, social security beneficiaries, traffic noise and lower social cohesion remained significantly associated with depression in multivariable analyses combining all neighbourhood factors (Table 2). In addition, higher home value and more water in the neighbourhood became significantly associated with depression in these multivariable analyses [OR (95% CI) = 1.29 (1.08–1.55) and OR (95%CI) = 1.13 (1.01–1.26)].

**Table 2.** Associations between neighbourhood characteristics and current diagnoses of depressive and anxiety disorders

	Depressive and/or anxiety disorder (*n* = 1783) *v.* controls (*n* = 1197)	Depressive disorder (*n* = 1275) *v.* controls (*n* = 1197)	Anxiety disorder (*n* = 1363) *v.* controls (*n* = 1197)
	OR (95% CI)	OR (95% CI)	OR (95% CI)
Urbanization grade			
Not urbanized	0.76 (0.49, 1.19)	0.74 (0.45, 1.22)	0.74 (0.46, 1.20)
Hardly urbanized	1.29 (0.83, 2.01)	1.48 (0.91, 2.40)	1.18 (0.73, 1.91)
Moderately urbanized	1.13 (0.81, 1.57)	1.10 (0.76, 1.59)	1.10 (0.77, 1.57)
Strongly urbanized	0.89 (0.68, 1.18)	0.99 (0.73, 1.34)	0.86 (0.63, 1.17)
Extremely urbanized	Reference	Reference	Reference
Socioeconomic neighbourhood			
Socioeconomic status score	0.88 (0.80, 0.97)**	0.88 (0.79, 0.98)*	0.88 (0.80, 0.97)**
Home value	1.06 (0.95, 1.19)	1.04 (0.92, 1.18)	1.09 (0.97, 1.23)
Social security beneficiaries	1.20 (1.08, 1.33)**	1.24 (1.11, 1.39)**	1.21 (1.09, 1.36)**
Immigrants	1.16 (1.00, 1.34)*	1.23 (1.05, 1.44)**	1.16 (1.00, 1.35)*
Physical neighbourhood			
Air pollution	1.15 (0.99, 1.33)	1.09 (0.93, 1.28)	1.21 (1.04, 1.41)*
Traffic noise	1.17 (1.05, 1.30)**	1.17 (1.03, 1.32)**	1.22 (1.09, 1.38)**
Green space	0.90 (0.81, 1.01)	0.89 (0.79, 1.01)	0.89 (0.79, 1.00)*
Water in neighbourhood	1.10 (1.00, 1.21)*	1.09 (0.99, 1.21)	1.12 (1.02, 1.24)*
Social neighbourhood			
Social cohesion	0.86 (0.79, 0.94)**	0.84 (0.76, 0.93)**	0.86 (0.78, 0.94)**
Safety	0.82 (0.70, 0.96)**	0.84 (0.71, 1.00)*	0.79 (0.67, 0.93)**
*Multivariable model*^a^	*N* = *2885*	*N* = *2393*	*N* = *2478*
Urbanization grade	0.78 (0.56, 1.10)	0.88 (0.61, 1.28)	0.79 (0.54, 1.13)
Socioeconomic status score	0.95 (0.80, 1.12)	1.04 (0.86, 1.25)	0.97 (0.81, 1.15)
Home value	1.31 (1.11, 1.54)**	1.29 (1.08, 1.55)**	1.37 (1.16, 1.62)**
Social security beneficiaries	1.30 (1.05, 1.61)*	1.41 (1.11, 1.79)**	1.38 (1.10, 1.75)**
Immigrants	0.99 (0.79, 1.23)	1.05 (0.83, 1.34)	0.96 (0.76, 1.22)
Air pollution	0.93 (0.74, 1.16)	0.87 (0.69, 1.12)	0.93 (0.73, 1.18)
Traffic noise	1.23 (1.07, 1.42)**	1.26 (1.08, 1.47)**	1.29 (1.11, 1.50)**
Green space	0.97 (0.83, 1.12)	0.94 (0.80, 1.12)	0.97 (0.83, 1.15)
Water in neighbourhood	1.13 (1.02, 1.25)*	1.13 (1.01, 1.26)*	1.16 (1.04, 1.29)**
Social cohesion	0.91 (0.80, 1.02)	0.86 (0.75, 0.99)*	0.93 (0.82, 1.06)
Safety	0.95 (0.76, 1.19)	1.10 (0.85, 1.41)	0.89 (0.69, 1.13)

Based on multilevel logistic regression analyses including municipality code as random intercept, adjusted for sex, age, education and household income. *p ⩽ 0.05; **p ⩽ 0.01. All continuous neighbourhood characteristics were standardized: OR are per 1 s.d. increase; socioeconomic status score: s.d. = 1.18, home value: s.d. = 70.5, social security beneficiaries: s.d. = 43.0, immigrants: s.d. = 15.0, road-, rail- and air-traffic noise: s.d. = 3.21, air pollution (PM_2.5_ absorbance): s.d. = 0.27, green space: s.d. = 22.6, water: s.d. = 6.21, safety: s.d. = 25.8, social cohesion: s.d. = 13.8.

a Urbanization grade was entered as a dichotomous variable (more or less than 1500 addresses/km^2^) in the multivariable model for ease of presentation.

Regarding anxiety, low neighbourhood socioeconomic status [OR (95% CI) = 0.88 (0.80–0.97)], more social security beneficiaries [OR (95% CI) = 1.21 (1.09–1.36)], more immigrants [OR (95%CI) = 1.16 (1.00–1.35)], high levels of air pollution [OR (95% CI) = 1.21 (1.04–1.41)], high levels of traffic noise [OR (95% CI) = 1.22 (1.09–1.38)], less green space [OR (95% CI) = 0.89 (0.79–1.00)], more water in the neighbourhood [OR (95% CI) = 1.12 (1.02–1.24)], lower social cohesion [OR (95% CI) = 0.86 (0.78–0.94)] and less safety [OR (95%CI) = 0.79 (0.67–0.93)] were significantly associated with anxiety disorders (Table 2, column 3; all *p* ⩽ 0.05). Of these factors, social security beneficiaries, traffic noise and water in the neighbourhood remained significantly associated with anxiety in multivariable analyses (Table 2). In addition, higher home value became significantly associated with anxiety in these multivariable analyses [OR (95% CI) = 1.37 (1.16–1.62)]. Thus, overall, not urbanization grade but rather socioeconomic, physical and social neighbourhood factors were associated with depression and anxiety, even after adjustment for covariates and other neighbourhood factors.

Additional sensitivity analyses only including subjects who lived at their home address for 2 years or longer (*n* = 2485) showed similar effect sizes for all neighbourhood factors that were statistically significant with the prevalence of depressive and/or anxiety disorder in Table 2 (ΔOR range, −6% to +3%). Also, analyses only including persons that did not report any relocations between baseline and follow-up measurement in 2006–2009 (*n* = 1986) resulted in comparable effect sizes (ΔOR range, −7% to + 6%). Overall, this suggests our findings seem rather stable and are not affected much by instability in home address around our assessment period. Additional sensitivity analyses including a more ‘clean’ control group without lifetime diagnoses of depression and anxiety (*n* = 2435 of which 652 controls) showed similar or even somewhat larger effect sizes (see online Supplementary Table 2). All earlier found associations with the prevalence of depressive and/or anxiety disorders remained statistically significant except the association for traffic noise [OR (95% CI) = 1.11 (0.96–1.27), *p* = 0.15] and water in the neighbourhood [OR (95% CI) = 1.07 (0.95–1.19), *p* = 0.26]. On the contrary, green space became significantly associated with lower prevalence of depression and/or anxiety disorders when using this ‘clean’ control group [OR (95% CI) = 0.84 (0.73–0.96), *p* = 0.01].

Previous research suggests multilevel data should include at least five cases per cluster (Clarke, 2008; McNeish & Stapleton, 2014). Thus, we performed sensitivity analyses only including subjects from municipalities with at least five persons (*n* = 2721). These sensitivity analyses performed for the variables that were significant in univariable analyses (Table 2, column 1) showed that odds ratios (OR) for all neighbourhood factors were similar (ΔOR ranging from −4% to 0%) in association with the prevalence of depressive and/or anxiety disorder. To be precise, effect sizes were as follows: socioeconomic status: OR (95% CI) = 0.88 (0.80–0.97); social security beneficiaries: OR (95% CI) = 1.20 (1.08–1.34); immigrants: OR (95% CI) = 1.13 (0.97–1.31); traffic noise: OR (95% CI) = 1.12 (0.99–1.27); water: OR (95% CI) = 1.08 (0.98, 1.19); social cohesion: OR (95% CI) = 0.85 (0.77–0.93); safety: OR (95%CI) = 0.81 (0.68–0.97). Thus, the exclusion of participants from municipalities with low sample size does not change our findings.

### Neighbourhood characteristics and the severity of depressive, anxiety and fear symptoms

Multilevel linear regression analyses with symptom severity confirmed that not urbanization grade, but rather socioeconomic, physical and social neighbourhood characteristics were significantly associated with depressive, anxiety and fear symptoms, also after adjusting for covariates (Table 3). A higher number of social security beneficiaries and lower levels of safety in the neighbourhood were associated with increased levels of all types of symptoms. In addition, lower socioeconomic status, more immigrants and more traffic noise in the neighbourhood were associated with increased depressive and anxiety symptoms. Lower social cohesion was associated with increased depressive and fear symptoms. Multivariable analyses combining all neighbourhood factors showed that these effects remained significant for social security beneficiaries and traffic noise with depressive and anxiety symptoms (Table 3). Also, more water became significantly associated with increased depressive symptoms, and higher home value became significantly associated with increased depressive and fear symptoms in these multivariable analyses.

**Table 3.** Associations between neighbourhood characteristics and the severity of depressive, anxiety and fear symptoms

	Depressive symptoms (IDS) *N* = 2941		Anxiety symptoms (BAI) *N* = 2945		Fear symptoms (FQ) *N* = 2945	
	*β*	95% CI	*β*	95% CI	*β*	95% CI
Urbanization grade						
Not urbanized	−0.19	−0.39, 0.01	−0.17	−0.37, 0.01	0.02	−0.16, 0.20
Hardly urbanized	0.07	−0.12, 0.25	0.02	−0.15, 0.20	0.11	−0.07, 0.28
Moderately urbanized	0.03	−0.11, 0.18	0.03	−0.12, 0.17	0.02	−0.12, 0.16
Strongly urbanized	−0.01	−0.13, 0.11	−0.0002	−0.12, 0.12	0.07	−0.06, 0.19
Extremely urbanized		Reference		Reference		Reference
Socioeconomic neighbourhood						
Socioeconomic status score	−0.06	−0.10, −0.02**	−0.04	−0.08, −0.001*	−0.02	−0.06, 0.02
Home value	−0.01	−0.06, 0.03	0.007	−0.04, 0.05	0.02	−0.02, 0.07
Social security beneficiaries	0.11	0.07, 0.16**	0.08	0.04, 0.13**	0.05	0.01, 0.09*
Immigrants	0.14	0.08, 0.20**	0.11	0.06, 0.17**	0.05	−0.01, 0.11
Physical neighbourhood						
Air pollution	0.02	−0.04, 0.08	0.05	−0.01, 0.11	0.01	−0.04, 0.07
Traffic noise	0.08	0.03, 0.13**	0.09	0.05, 0.14**	0.01	−0.03, 0.06
Green space	−0.03	−0.08, 0.02	−0.02	−0.06, 0.03	−0.01	−0.05, 0.04
Water in neighbourhood	0.03	−0.01, 0.07	0.02	−0.02, 0.06	0.02	−0.02, 0.06
Social neighbourhood						
Social cohesion	−0.05	−0.09, −0.01**	−0.04	−0.08, 0.003	−0.05	−0.08, −0.01*
Safety	−0.11	−0.17, −0.04**	−0.09	−0.15, −0.03**	−0.05	−0.11, 0.002*
*Multivariable model*^a^		*N* = 2847		*N* = 2851		*N* = 2851
Urbanization grade	−0.01	−0.15, 0.13	−0.009	−0.15, 0.13	−0.002	−0.14, 0.14
Socioeconomic status score	0.03	−0.04, 0.10	0.03	−0.04, 0.10	0.01	−0.06, 0.08
Home value	0.08	0.001, 0.15*	0.07	−0.001, 0.13	0.09	0.02, 0.15**
Social security beneficiaries	0.14	0.05, 0.23**	0.11	0.02, 0.21*	0.07	−0.02, 0.16
Immigrants	0.05	−0.05, 0.14	0.05	−0.04, 0.14	0.02	−0.08, 0.11
Air pollution	−0.09	−0.18, 0.009	−0.04	−0.13, 0.05	−0.07	−0.16, 0.02
Traffic noise	0.12	0.06, 0.18**	0.12	0.06, 0.17**	0.04	−0.02, 0.09
Green space	0.02	−0.04, 0.09	0.04	−0.02, 0.10	0.02	−0.04, 0.09
Water in neighbourhood	0.04	0.00002, 0.08*	0.03	−0.01, 0.07	0.03	−0.02, 0.07
Social cohesion	−0.03	−0.08, 0.02	−0.01	−0.07, 0.04	−0.04	−0.09, 0.01
Safety	−0.05	−0.15, 0.04	−0.06	−0.15, 0.04	−0.05	−0.14, 0.04

Based on multilevel linear regression analyses including municipality code as random intercept, adjusted for sex, age, education and household income. *p ⩽ 0.05; **p ⩽ 0.01. All symptom scales and continuous neighbourhood characteristics were standardized. Fixed parameter estimate (*β*) is per 1 s.d. increase; socioeconomic status score: s.d. = 1.18, home value: s.d. = 70.5, social security beneficiaries: s.d. = 43.0, immigrants: s.d. = 15.0, road-, rail- and air traffic noise: s.d. = 3.21, air pollution (PM_2.5_ absorbance): s.d. = 0.27, green space: s.d. = 22.6, water: s.d. = 6.21, safety: s.d. = 25.8, social cohesion: s.d. = 13.8.

a Urbanization grade was entered as a dichotomous variable (more or less than 1500 addresses/km^2^) in the multivariable model for ease of presentation.

## Discussion

This cross-sectional study (*N* = 2980) showed that not urbanization grade, but rather socioeconomic, physical and social characteristics of the neighbourhood were associated with the presence and severity of depression and anxiety, independent of individual indicators of socioeconomic status.

These findings add to the body of evidence that characteristics of the neighbourhood seem to be associated with depression and anxiety (Evans, 2003; Kim, 2008; Mair *et al.* 2008; Diez Roux & Mair, 2010; Gariepy *et al.* 2014). Overall, our results were consistent across different assessment methods (DSM-based interviews *v.* symptom scales). Contrary to previous research (de Vries *et al.* 2016) this might suggest that the neighbourhood context is relevant for both clinical diagnoses of depression and anxiety as well as more general reflections of negative mood. Our findings may implicate that affective disorders are not related to population density in the neighbourhood, but rather with the quality of the neighbourhood. This may be surprising in light of previous research which consistently showed urbanicity to be associated with increased prevalence of depressive and anxiety disorders (Peen *et al.* 2010; Zijlema *et al.* 2015) and other types of psychiatric disorders, such as schizophrenia and psychosis (Krabbendam & van Os, 2005). On the other hand, our finding is in line with a literature review (Judd *et al.* 2002) showing that a variety of sociodemographic factors, such as unemployment, low socioeconomic status and poor social support, were stronger predictors of affective disorders than urbanization level, and that many previous studies failed to take these factors into account (Judd *et al.* 2002).

In our study, a high number of social security beneficiaries, high home values, high levels of road- rail- and air-traffic noise (measured as Lden in dB), more water in the neighbourhood and lower social cohesion were most consistently associated with depression and anxiety, independent of covariates and other neighbourhood factors. Social security beneficiaries are an indicator of long-term unemployment and socioeconomic deprivation of residents in the area, factors which have previously been associated with poor mental health and depression (Kim, 2008; Annequin *et al.* 2015; Klijs *et al.* 2016). Moreover, we showed traffic noise (Lden in dB) to be associated with depression and anxiety, independent of air pollution (measured as PM_2.5_ absorbance) and urbanization grade, whereas many previous studies failed to adjust for these characteristics (Tzivian *et al.* 2015). Although causal processes remain speculative, it could be that traffic noise increases levels of stress (Arnsten & Goldman-Rakic, 1998), subsequently affecting both physical and mental health (Recio *et al.* 2016; Chen *et al.* 2017). Our data did not allow one to distinguish between nocturnal and daily noise exposure, but likely these are highly correlated. High levels of traffic noise may possibly result in increased arousal (Griefahn *et al.* 2008) and concentration problems (Bodin *et al.* 2015) but also in more sleep disturbances, which have consistently been associated with higher risks of depressive and anxiety disorders, also in the NESDA sample (van Mill *et al.* 2010). According to the WHO guidelines, outdoor noise levels higher than 45 decibels could result in sleep disturbance effects (Berglund *et al.* 2000). An alternative explanation is that traffic noise is a proxy of other forms of neighbourhood deprivation, such as annoyance (Ragettli *et al.* 2015*a*) and overcrowding (O'Campo *et al.* 2009). Our finding that lower social cohesion is associated with an increased risk of depression is in line with previous research (Elliott *et al.* 2014), although most of these studies did not use objectively attained census data and did not account for other relevant neighbourhood correlates (Mair *et al.* 2008; Ruijsbroek *et al.* 2016). The social neighbourhood could directly influence psychopathology, or indirectly through for example other person-level characteristics such as social relationships (Cohen & Wills, 1985; Helgeson, 2003). Also, research suggests that social neighbourhood cohesion might mediate the relationship between income deprivation and mental health (Fone *et al.* 2014).

Our findings that higher home values and more water in the area are associated with poor mental health are surprising in light of previous research showing beneficial health effects of high neighbourhood income (Klijs *et al.* 2016) and living close to water (White *et al.* 2013; Grellier *et al.* 2017). Regarding our result for water, one possible explanation is our assessment method of blue space, which is different from other previous studies that measured the percentage of blue space by calculating a ‘buffer’ around the centroid of the postal code area (e.g. de Vries *et al.* 2016). Another explanation is that perhaps blue space is not a very good indicator of a ‘nature rich’ environment for the Dutch situation, because the percentage of water appeared to be highest in highly population areas. There are quite some cities with canals (e.g. Amsterdam) closely located to the Dutch coast, hence leading to positive effects of more water with higher risks of affective disorders. Consequently, the concept of ‘blue space’ in The Netherlands may not be exactly comparable to the concept in other countries, and therefore its findings should be interpreted carefully. Also, it is known that house prices in Dutch cities can be extremely high because of high demand and popularity. This might explain the association between high home values with higher risks of depression and anxiety (in multivariable analyses), and the positive correlations of high home values with air pollution and traffic noise (online Supplementary Table 1). High home values in the neighbourhood are likely to be a marker of higher socioeconomic status (*r* = 0.66; online Supplementary Table 1) and less social security beneficiaries (*r* = −0.64; online Supplementary Table 1), and this is found in the city center of Amsterdam. So, when interpreting these associations, it is important to realize that the Netherlands is amongst the most densely populated countries in the world. This may have introduced some specific characteristics. In other – less dense populated – areas, such associations could be different.

Although our study is cross-sectional of nature and therefore causality cannot be inferred, it would be adequate to provide some speculations regarding the pathways linking the neighbourhood to affective disorders. The social causation hypothesis asserts that poor neighbourhoods increase the risk of subsequent affective disorders (Mossakowski, 2014). The neighbourhood might be linked with mental health through behavioural and psychological stressors (e.g. social activity or overcrowding; Evans, 2003) or biological regulation pathways (e.g. increased inflammation; Bird *et al.* 2010). Other proposed mediators between neighbourhoods and affective disorders could be unhealthy lifestyles (Saelens *et al.* 2003; Lorenc *et al.* 2012), poor perception and satisfaction of the neighbourhood (Leslie & Cerin, 2008), poor sense of control (Thoits, 2011), personality vulnerabilities such as neuroticism (Blair *et al.* 2014) or the actual environmental exposure (i.e. some persons ‘use’ their neighbourhood more than others). However, a reverse link between environmental factors and affective disorders is also possible. The selection/drift hypothesis posits that poor mental health can inhibit socioeconomic attainment and lead people to drift into lower social class, resulting in (the maintenance of) less favourable environments (Pickett & Pearl, 2001; Mossakowski, 2014). So, in sum, a bidirectional link is not unlikely. Deprived neighbourhoods may affect the resident's mental health and, reversely, the resident's health status may affect the state or selection of the neighbourhood. Future studies should shed light on potential causal and mediating pathways between neighbourhoods and affective disorders, and on how such a link could be intervened on in order to stimulate global mental health.

Strong aspects of this study are the large sample size, the objective measurement of neighbourhood factors using census data, the inclusion of both validated psychiatric interviews (Robins *et al.* 1988) and symptoms scales (van Zuuren, 1988; Beck & Steer, 1990; Rush *et al.* 1996) for the assessment of depression and anxiety, and the adjustment for other neighbourhood-level and person-level confounders. Next to these important strengths, there are also several methodological considerations to our epidemiological study. First, our study is limited by the cross-sectional nature. However, we feel that generating a clear overview of the neighbourhood factors cross-sectionally associated with mental health is a most valuable first step in the neighbourhood and mental health research. It would be very useful to follow our study up with longitudinal approaches in which neighbourhood characteristics are for example linked with the development of mental health problems over time. Thus, future prospective studies and population-level experimental studies might shed more light on whether deprived neighbourhoods are contributors, correlates or consequences of affective disorders. These future studies are highly recommended to take a broad scope of the neighbourhood and select more than one factor and to place studies within a plausible theoretical framework (Schofield & Das-Munshi, 2017). Second, it could be that the use of more sophisticated Global Positioning System (GPS) tracking measures for the assessment of residential living space conceals somewhat different findings than the use of linkage through postal code areas, e.g. because environmental exposure at home and work could be combined (Ragettli *et al.* 2015*b*). However, GPS methods may represent a step backward in terms of biases related to selective daily mobility and causal inferences (Chaix *et al.* 2013). Moreover, GPS methods are more costly, whereas the use of existing registration systems is easier, highly feasible and in line with various previous cohort studies (Mair *et al.* 2008; Zijlema *et al.* 2015; de Vries *et al.* 2016; Klijs *et al.* 2016; Chen *et al.* 2017). Third, we aggregated neighbourhood data to average values for four-digit postal code areas as this was the most precise information available of our respondents, and this method is in accordance with many previous Dutch studies (Wittebrood, 2000; 2004; Van Wilsem, 2003; Bernasco & Nieuwbeerta, 2005; Nieuwbeerta *et al.* 2008). Aggregation results in less variation in values for air pollution (as indicated by PM_2.5_ absorbance) and traffic noise (as indicated by Ldn in dB) that were estimated at a lower geographical level, i.e. residential addresses. However, our geodataset including all 4766 postal code areas of the Netherlands allowed us to compare distributions for air pollution and traffic noise data between four-digit and six-digit postal code areas and these appeared comparable [air pollution, mean (s.d.) = 1.18(0.22) *v.* 1.27(0.25); traffic noise, median (IQR) = 54 (51–56) *v.* 54 (51–59)]. In combination with our significant findings for noise with depression and anxiety and for pollution with anxiety, this seems to suggest that there is still enough variation in our variables to detect group differences. Fourth, as previously mentioned, the Netherlands is a very densely populated country (±483 residents/km^2^ in 2006; CBS, 2006*e*); hence, future research could test whether our results can be generalized to other countries.

Nonetheless, to our knowledge, this is the first large-scale study that assessed multiple objectively attained socioeconomic, physical and social neighbourhood factors in relation to both the presence and severity of depression and anxiety disorders, while adjusting for individual indicators of socioeconomic status.

It suggests that socioeconomic, physical and social neighbourhood characteristics are associated with affective disorders, implying the relevance of studying the psychological impact of neighbourhood quality.

## Supplementary material

For supplementary material accompanying this paper visit https://doi.org/10.1017/S0033291718000612. click here to view supplementary material
