# Supplementary material for: Not urbanization level but socioeconomic, physical and social neighbourhood characteristics are associated with presence and severity of depressive and anxiety disorders
Source: Psychol Med. 2018 Mar 15;49(1):149–61. doi: 10.1017/S0033291718000612 (PMC6316373; doi:10.1017/S0033291718000612)
Supplement: Supplementary file 1 [file S0033291718000612sup001.zip › S0033291718000612sup001/SupplTable1.docx]

| **Supplementary Table 1. Intercorrelations of neighbourhood factors (N=2980)** | | | | | | | | | | |  | |
| --- | --- | --- | --- | --- | --- | --- | --- | --- | --- | --- | --- | --- |
|  |  | **Urbanicity** | **Socioeconomic neighbourhood** | | | | **Physical neighbourhood** | | | | **Social neighbourhood** | |
|  | | Urbanization grade | Socioeconomic status score | Home value | Social security beneficiaries | Immigrants | Air pollution | Traffic noise | Green space | Water in neighbour-  hood | Social cohesion | Safety |
| Urbanization grade | | 1 | -.41** | -.22** | .54** | .79** | .70** | .52** | -.75** | .40** | -.49** | -.74** |
| Socioeconomic status score | |  | 1 | .66** | -.83** | -.47** | -.07** | -.13** | .38** | -.04* | .39** | .44** |
| Home value | |  |  | 1 | -.64** | -.17** | .25** | .12** | .25** | .01 | .29** | .27** |
| Social security  beneficiaries | |  |  |  | 1 | .61** | .12** | .13** | -.42** | .07** | -.41** | -.59** |
| Immigrants | |  |  |  |  | 1 | .63** | .47** | -.54** | .26** | -.30** | -.69** |
| Air pollution | |  |  |  |  |  | 1 | .66** | -.56** | .29** | -.34** | -.59** |
| Traffic noise | |  |  |  |  |  |  | 1 | -.47** | .26** | -.19** | -.37** |
| Green space | |  |  |  |  |  |  |  | 1 | -.22** | .54** | .67** |
| Water in neighbourhood | |  |  |  |  |  |  |  |  | 1 | -.11** | -.27** |
| Social cohesion | |  |  |  |  |  |  |  |  |  | 1 | .61** |
| Safety | |  |  |  |  |  |  |  |  |  |  | 1 |

Based on Spearman correlations for urbanization grade and Pearson correlations for other neighbourhood variables. Urbanization grade: ‘not urbanized’ is the reference group. * p<0.05; ** p<0.001
