# Supplementary material for: Not urbanization level but socioeconomic, physical and social neighbourhood characteristics are associated with presence and severity of depressive and anxiety disorders
Source: Psychol Med. 2018 Mar 15;49(1):149–61. doi: 10.1017/S0033291718000612 (PMC6316373; doi:10.1017/S0033291718000612)
Supplement: Supplementary file 1 [file S0033291718000612sup001.zip › S0033291718000612sup001/SupplTable2_cleancontrols.docx]

**Supplementary 2. Associations between neighbourhood characteristics**

**and current diagnoses of depressive and anxiety disorders**

**when using a more ‘clean’ control group without lifetime diagnoses of**

**depressive and anxiety disorders**

|  | **Depressive and/or anxiety disorder (n=1783) vs controls without lifetime diagnoses (n=652)** | |
| --- | --- | --- |
|  | **OR (95%CI)** | ***P*** |
| **URBANIZATION GRADE** |  |  |
| Not urbanized | 0.70 (0.40, 1.22) | 0.21 |
| Hardly urbanized | 1.47 (0.83, 2.61) | 0.19 |
| Moderately urbanized | 1.29 (0.85, 1.96) | 0.23 |
| Strongly urbanized | 0.98 (0.70, 1.38) | 0.92 |
| Extremely urbanized | Reference |  |
| **SOCIOECONOMIC**  **NEIGHBOURHOOD** |  |  |
| Socioeconomic status score | 0.85 (0.76, 0.95)** | 0.005 |
| Home value | 1.03 (0.90, 1.18) | 0.67 |
| Social security beneficiaries | 1.32 (1.16, 1.51)** | <0.001 |
| Immigrants | 1.24 (1.04, 1.49)* | 0.02 |
| **PHYSICAL NEIGHBOURHOOD** |  |  |
| Air pollution | 1.19 (1.00, 1.41) | 0.06 |
| Traffic noise | 1.11 (0.96, 1.27) | 0.15 |
| Green space | 0.84 (0.73, 0.96)** | 0.01 |
| Water in neighbourhood | 1.07 (0.95, 1.19) | 0.26 |
| **SOCIAL NEIGHBOURHOOD** |  |  |
| Social cohesion | 0.82 (0.73, 0.91)** | <0.001 |
| Safety | 0.73 (0.60, 0.88)** | 0.001 |

Based on multilevel logistic regression analyses including municipality code as random

intercept, adjusted for sex, age, education and household income. *p ≤ 0.05; **p ≤ 0.01.

All continuous neighbourhood characteristics were standardized: OR are per 1 SD increase;
socioeconomic status score: SD=1.18, home value: SD=70.5, social security beneficiaries:
SD=43.0, immigrants: SD=15.0, road-, rail- and air traffic noise: SD=3.21, air pollution

(blackness of carbon filters): SD=0.27, green space: SD=22.6, water: SD=6.21, safety:

SD=25.8, social cohesion: SD=13.8.
